# Arthroscopy in specific joint pathologies of dogs and potential for application in wildlife species

**DOI:** 10.4142/jvs.26015

**Published:** 2026-07-06

**Authors:** Young Deok Suh, Soeun Ryu, Do Na Lee, Seong Chan Yeon

**Affiliations:** 1 Department of Veterinary Clinical Sciences, College of Veterinary Medicine and Research Institute for Veterinary Science, Seoul National University, Seoul 08826, Korea.; 2 Seoul Wildlife Center, Seoul National University, Seoul 08826, Korea.

**Keywords:** Minimally invasive surgical procedures, arthropathies, orthopedic procedures, veterinary medicine, treatment outcome

## Abstract

**Importance:**

Arthroscopy has become an essential minimally invasive technique for both diagnostic and surgical applications in veterinary medicine. However, despite its established clinical utility in domestic small animals, particularly for the evaluation and management of arthropathies in major joints of dogs, its application in wildlife species remains limited. Expanding the use of arthroscopy in wildlife species may address important gaps in the diagnosis and management of arthropathies across diverse taxa.

**Observations:**

This narrative review demonstrates that arthroscopy is extensively utilized in small animals, especially dogs, for both diagnostic and therapeutic management of major joint disorders, supported by continuous developments in instrumentation and procedural techniques. In contrast, arthroscopic applications in wildlife species are largely confined to isolated case reports, with considerable variability in anatomical feasibility, surgical approaches, and clinical outcomes, leading to a limited understanding of disease pathophysiology, treatment options, and clinical outcomes in non-domestic species. This disparity highlights a substantial gap in the evidence base regarding the use of arthroscopy across non-domestic species.

**Conclusions and Relevance:**

Arthroscopy provides a versatile and effective modality in veterinary orthopedics, with well-established applications in canine patients. However, its use in wildlife medicine remains limited and inconsistent. Continued refinement of arthroscopic equipment and techniques, along with the accumulation of clinical experience, may facilitate broader adoption in wildlife species. This review provides a timely overview of current practices and highlights clinically relevant gaps, supporting future research and potential clinical application in non-domestic species.

## INTRODUCTION

Since the 1950s, arthroscopy has evolved into a standard modality in human orthopedic surgery, largely replacing major joint arthrotomies [1]. In veterinary medicine, arthroscopy was first adopted in equine surgery in the 1970s, and subsequently introduced to dogs in 1978 [1,2,3,4]. The relatively delayed adoption in small animals was due to limitations related to joint size, instrument availability, and equipment costs, rather than a lack of clinical relevance [1,2,3].

Advances in optics, light sources, and instrument miniaturization have enabled widespread use of arthroscopy in major joints of dogs and cats [5,6]. Currently, arthroscopy accounts for more than half of all minimally invasive procedures performed by small animal surgeons certified by the American College of Veterinary Surgeons [3]. Beyond reduced invasiveness, arthroscopy has improved diagnostic accuracy and early clinical decision making by enabling real-time assessment of intra-articular structures and cartilaginous, ligamentous, and synovial pathologies [5,6].

In contrast to domestic animals, reports of arthroscopic procedures in wildlife species remain limited [7]. Most published accounts involve isolated descriptions of diagnostic or surgical procedures in both captive and free-ranging animals. Consequently, clinical understanding of pathophysiology and treatment options for arthropathies in wildlife species remains obscure [7].

This review summarizes the current practice of arthroscopy in small animals, with particular emphasis on instrumentation, equipment, and diagnostic and surgical applications in major canine joints. In addition, it synthesizes the limited but emerging literature addressing arthropathies and arthroscopic procedures in wildlife species to explore potential translational clinical applications of arthroscopy as a minimally invasive modality for improving *in vivo* diagnosis and management of arthropathies across diverse species and highlight clinically relevant knowledge gaps.

## METHODS

A literature review was undertaken to compile relevant information. Data were obtained through a literature search of textbooks and electronic databases including PubMed, Google Scholar, and the National Institutes of Health (publications up to December 2025). The search strategy utilized both single terms and multiple keyword combinations of “arthroscopy,” “orthopedics,” “domestic small animals,” “dog,” “wildlife species,” and additional subject- specific phrases. Given the limited volume of wildlife arthroscopy literature, this review was conducted as a narrative review rather than a systematic review, with single-case reports and small case series included when clinically relevant. The review was restricted to English-language peer-reviewed articles. This narrative review was conducted in accordance with SANRA (Scale for the Assessment of Narrative Review Articles) guidelines.

## OBSERVATIONS

### Section 1. Arthroscopic instrumentation and equipment

#### Arthroscopes, cannulas

##### 1) Telescopes

In small animal arthroscopy, 2.4–2.7 mm diameter rigid arthroscopes are common; 2.4 mm systems are preferred for their optics and suitability for smaller joints; needle arthroscopes (1–2 mm diameter) are increasingly being utilized [8,9,10].

Smaller diameter arthroscopes (1.9 mm) are primarily reserved for smaller joints, whereas 4.0 mm arthroscopes are reserved for equine procedures and stifle arthroscopy in giant dog breeds [10,11]. A 30° forward-oblique telescope remains the most commonly used, as it provides a broad field of view through axial rotation without major repositioning (Table 1).

**Table 1:** Common telescopes used in veterinary arthroscopy^a^

Arthroscope type	Diameter (mm)	Working length (cm)	Viewing angle (°)	Key characteristics
Rigid Hopkins rod-lens arthroscope	4.0	12 (short)/18 (long)	30	Primarily reserved for usage in large joints due to large diameter; mostly used in large animal arthroscopy
Rigid, rod-lens endoscopic telescope with steerable direction of view	4.0	18	15–90 (variable)	Steerable rod-lens telescope with a variable viewing angle (15–90°), enabling dynamic intra-articular visualization
Rigid Hopkins rod-lens arthroscope	2.7	11 (short)/18 (long)	30	Historically the most widely used arthroscope; the long arthroscope used as a multipurpose arthroscope (MPRT), whereas the short arthroscope is more robust and suited for smaller joints but optically inferior to the 2.4 mm arthroscope
Rigid Hopkins rod-lens arthroscope	2.4	11	30	Current standard arthroscope in small animal practice; short working length and improved blunt obturator design enhance maneuverability and ease of placement in confined joint spaces
Rigid Hopkins rod-lens arthroscope	1.9	6.5/10	30	Small-diameter arthroscope with limited optical performance; suitable for small joints (eg. radiocarpal / tibiotarsal joints) and extremely small patients (eg. puppies, kittens, and rabbits)

^a^From reference [8].

##### 2) Telescopic sheaths

Telescopic sheaths protect the telescope and enable continuous joint irrigation, with contemporary designs incorporating stop cocks with Luer-lock connectors for controlled fluid inflow. Sheaths are supplied with either sharp trocars or blunt obturators, with blunt obturators preferred to minimize the risk of iatrogenic cartilage damage during portal establishment [11,12]. Locking mechanisms secure the telescope to the sheath, protect the distal lens, and maintain a watertight seal for consistent irrigation [8,11].

##### 3) Operative cannulas

Operative cannulas provide stable access for instrument switching, but may limit instrument size and tissue retrieval. Cannulas are supplied with sharp trocars or blunt obturators and lack valves or stopcocks, allowing passive fluid egress when instruments are absent [8,11]. Accurate placement under direct arthroscopic visualization is essential to minimize iatrogenic intra-articular damage [12].

##### 4) Egress cannulas

Egress cannulas serve as dedicated outflow portals for irrigation and help prevent excessive intra-articular pressure. Commonly used cannulas incorporate multiple side holes and a stopcock for unobstructed and regulated drainage [8,11]. Placement is performed under direct arthroscopic guidance to ensure optimal positioning and function [11,12].

#### Hand instruments

##### 1) Arthroscopic hand instrumentation

Common arthroscopic hand instruments include rongeurs, grasping forceps, microfracture chisels, curettes, hook probes, arthroscopic knives, and meniscal resection forceps [8,11]. Instrument selection is dictated by joint anatomy, lesion characteristics, and the need to balance precise lesion removal with soft tissue preservation.

##### 2) Surgical instruments and supplies

Standard surgical instruments required for arthroscopy are listed in Table 2. Complete open surgery sets should be available for immediate conversion to arthrotomy when indicated [11].

**Table 2:** Standard surgical instruments/supplies required for arthroscopy^a^

Category	Specific items
Blades	No. 11 scalpel blades
Needles	20-gauge 1 inch and 1.5-inch hypodermic needles
	20-gauge 2.5 inch or 3.0-inch spinal needles
Syringes	3-cc syringes
	12-cc syringes
Irrigation	3-way stopcock
	1-L containers of Normal saline or Lactated Ringer’s solution
	IV administration sets
	IV extension sets
	Pressure cuff for fluid containers
Hand instruments	Curved mosquito hemostats without teeth
	Curved mosquito hemostats with teeth
	Basic orthopedic surgery instrument sets

^a^From reference [8].

##### 3) Additional instruments

Certain procedures require specialized instrumentation. For instance, the Leipzig stifle distractor, enlarges the stifle joint space by applying cranial force on the tibial plateau, and is frequently employed during meniscectomies to improve visualization and instrument access [8,13,14].

#### Power instruments

##### 1) Power shavers

Power shavers enhance procedural efficiency by creating uniformly smooth debridement surfaces while minimizing postoperative debris [11,15]. Available blades include burrs and full-radius cutters designed for bone, cartilage, and soft tissue resection; all blades are hollow and cannulated to permit suction-assisted debris removal [15]. Disposable blades are recommended for sharpness retention, cost-effectiveness, and autoclave compatibility [8]. Shaver activation is controlled by hand or foot pedals, with foot control preferred to minimize unintended handpiece movement. Both two-pedal and three-pedal systems are available, with the latter preferred because it includes a center pedal for additional oscillation [8,15]. Optimal shaver performance depends on balanced irrigation to prevent debris accumulation and impaired visualization.

##### 2) Radiofrequency, electrocautery

Radiofrequency instruments are used for resection, vaporization, and coagulation of vascular and intra-articular soft tissues [8,11]. Thermally regulated bipolar systems designed for arthroscopy (VAPR) provide enhanced precision and thermal safety, while standard monopolar units, used with thermoregulated tips, are less suitable for ablation of larger tissue volumes [8,11].

#### Irrigation

Continuous irrigation is essential for adequate joint distension and visualization. Normal saline or lactated Ringer’s solution are most commonly used, with the latter preferred for reduced chondrocyte cytotoxicity [16]. Gravity flow, controlled through IV set controls and cannula stopcocks, remains the most practical method in clinical settings [17]. Maintaining a high-flow, low-pressure system through minimizing egress resistance is critical for maintaining clear visualization while minimizing periarticular fluid accumulation [8,11,17].

#### Imaging equipment

##### 1) Video system tower

A complete video endoscopy system forms the foundation of arthroscopic procedures and includes a minimum of a video camera, monitor, and light source on a mobile cart or cabinet (Fig. 1) [17]. Power shavers and radiofrequency units may be integrated into the tower to improve workflow efficiency, particularly during complex or prolonged procedures [8,17].

**Fig. 1:**
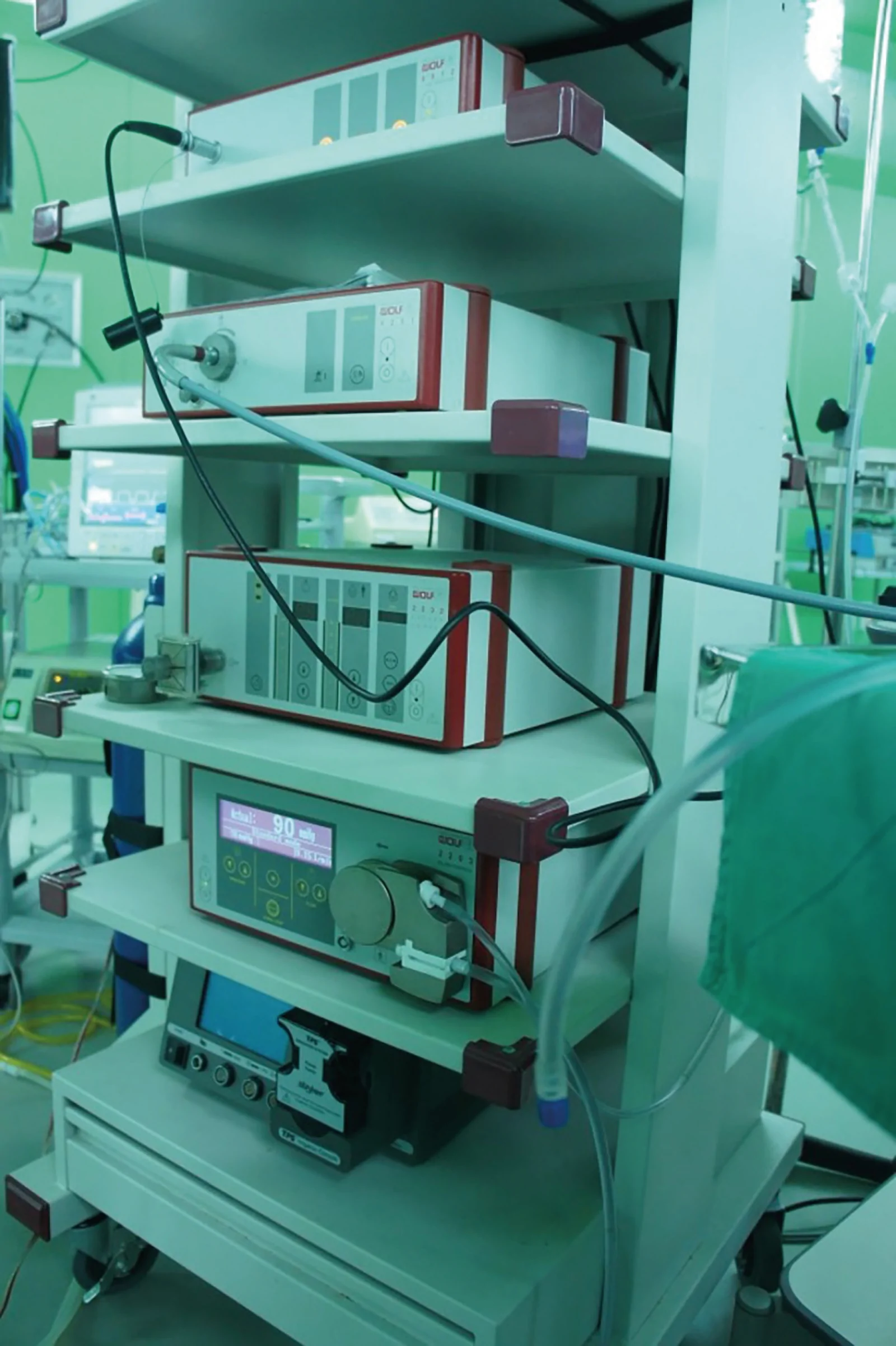
Basic endoscopy system^a^ assembled on a mobile cart or cabinet. ^a^Photograph provided by the corresponding author.

##### 2) Video camera

Dedicated arthroscopy cameras are fundamental for proper intra-articular visualization [17]. The camera head mounts directly onto the arthroscope eyepiece, creating an integrated imaging unit [17]. Contemporary lightweight, compact single-chip cameras provide sufficient image quality for most clinical procedures, while three-chip systems offer enhanced resolution and tissue color differentiation primarily for research applications and the acquisition of publication-quality images [8,17].

##### 3) Video monitor

High-resolution multi flat-screen monitors facilitate accurate visualization during arthroscopy. A common setup includes two monitors—one on the tower and another on the wall—to optimize visualization [8,11,17]. Wireless transmission systems further enhance operating room flexibility by enabling flexible monitor placement during complex procedures [17].

##### 4) Light source

Adequate arthroscopic visualization depends on a reliable high-intensity light source coupled with a fiber-optic cable. Modern systems commonly utilize diode-based light sources, which provide improved illumination with substantially extended service life [8,17]. Flexible fiberoptic cables connect the light source to the telescope, with interchangeable adapters ensuring compatibility between different endoscopic systems [17].

### Section 2. General technique

#### Patient positioning

Appropriate patient positioning facilitates optimal intra-articular access, visualization, and procedural efficiency, with representative positions summarized in Table 3 [18].

**Table 3:** Patient positioning for arthroscopy of the six joints^a^

Joint	Patient positioning	Preparation and notes
Elbow	Lateral recumbency	Medial aspect clipped, aseptically prepared, and draped
Shoulder	Lateral recumbency (for unilateral procedures)	Shoulder extended to 160° between scapula and humerus
	Dorsal recumbency (for bilateral procedures)	Forelimbs suspended for bilateral procedures
		Site clipped, prepared aseptically, draped
		Draping to allow patient rolling for bilateral procedures
Radiocarpal	Dorsal recumbency	Forelimb extended caudally for dorsal recumbency
	Lateral recumbency	Forelimb kept in normal standing angle and externally rotated
		Site clipped, prepared aseptically, draped
Stifle	Dorsal recumbency	Limb holder used to apply valgus/varus stress for better visualization
		Cranial aspect clipped, prepared aseptically, draped
Coxofemoral	Dorsal recumbency	Affected limb suspended
		Prepared and draped as for triple/double pelvic osteotomy
Tarsal	Dorsal recumbency	Hindlimbs extended caudally, abducted/adducted for joint access
		Site clipped and aseptically prepared

^a^From reference [18].

#### Portal placement

Portal locations for diagnostic and surgical arthroscopy are well established, and the following general principles are applicable to portal placements across all joints [18].

##### 1) Telescope portals

After joint palpation and anatomical landmark identification, a 20-gauge hypodermic needle is inserted, with aspiration of synovial fluid confirming intra-articular placement, followed by joint distention with sterile crystalloid solution [11,18]. A small stab incision through the skin, subcutaneous fascia, and joint capsule, allows insertion of the cannula with a blunt obturator into the joint [11,18]. The cannula is seated by palpating the obturator along articular contours and bony margins to minimize iatrogenic cartilage damage [11,18]. The obturator is removed, the telescope inserted, and irrigation is initiated to maintain visualization. The initial placement needle may be temporarily retained for fluid egress [11,18].

##### 2) Operative portals

Portal positioning is confirmed by arthroscopic visualization of a hypodermic needle placed at the proposed site and adjusted to obtain an optimal approach angle to the target lesion [17,18]. A small stab incision is made, and the operative portal is created by blunt dissection or by operative cannula insertion [17,18].

##### 3) Triangulation

Triangulation refers to the geometric alignment of the telescope and operative instrument forming two sides of a triangle, with the intra-articular lesion at the apex and the skin at the base [11,18]. A proper convergence angle is essential, as excessively narrow or wide angles compromise instrument controllability and procedural efficiency [11,18].

### Section 3. Diagnostic and surgical arthroscopy of canine forelimb

The most common diagnostic and surgical indications for arthroscopy in major canine joints of the forelimb are summarized in Table 4 [5].

**Table 4:** Major indications and potential complications of arthroscopy in canine forelimb joints^a^

Joints	Major indications	Potential complications
Shoulder	OCD	Instrument or telescope damage
	Bicipital tenosynovitis / tendon injuries	Articular cartilage injury
	Shoulder instability	Peri-articular soft tissue injury
	Incomplete fusion of the caudal glenoid ossification center	Bone fragment migration
	Reduction and stabilization of a supraglenoid tuberosity avulsion	Intra articular bleeding or periarticular fluid accumulation
	Diagnostic examination	Infection
	Septic arthritis	Nerve injury
Elbow	OCD	Instrument or telescope damage
	Fragmented medial coronoid process	Articular cartilage injury
	Ununited anconeal process	Peri-articular soft tissue injury
	Fractures of the humeral condyle and closed reduction and fixation	Bone fragment migration
	Diagnostic examination	Intra articular bleeding or periarticular fluid accumulation
	Septic arthritis	Infection
		Nerve injury
Radiocarpus	Evaluation of ligamentous damage	Instrument or telescope damage
	Intra-articular fractures, and removal of small bone chips	Articular cartilage injury
	Carpus instability	Peri-articular soft tissue injury
	Diagnostic examination	Bone fragment migration
	Septic arthritis	Intra articular bleeding or periarticular fluid accumulation
		Infection
		Nerve injury

OCD, osteochondritis dissecans.

^a^From reference [5].

### Arthroscopy of the elbow joint

#### Diagnostic arthroscopy of the elbow joint

##### 1) Medial coronoid process disease (MCPD)

*Free medial coronoid process fragments*, of varying sizes are commonly identified along with cartilage damage, although fragment size does not correlate with lesion severity [19,20,21]. *Fixed fragments* typically present as linear cartilage lesions including defects, chondromalacia, or mineralization [19,20,21,22,23,24,25,26]. *Chondromalacia*, graded using the modified Outerbridge system (Grades I–V) frequently affects the coronoid process and adjacent articular surfaces [19,20,21,22,23,24,25,26]. *Characteristic “glaciation” patterns*—linear cartilage wear extending from normal cartilage to exposed subchondral bone—are observed on Grade V lesions and exposed subchondral bone, predominantly on the ulna and the humeral condyle [19,25].

##### 2) Osteochondritis dissecans (OCD)

OCD lesions appear as raised, blistered, fibrillated, or fragmented cartilage surfaces with irregular full thickness margins mostly located on the medial margin and the highest portion of the medial humeral condylar ridge [19,27]. In clinical experience, OCD lesions frequently coexist with MCPD humeral condylar chondromalacia lesions, often demonstrating corresponding severity [19,27,28]. Distinction between OCD and MCPD-related chondromalacia is facilitated by the absence of the feathered or linear wear patterns characteristic of MCPD, a differentiation that is critical for an accurate diagnosis [19,27,28].

##### 3) Ununited anconeal process (UAP)

The cleavage width between the anconeal process fragment and the ulna varies according to fragment instability, ranging from a tightly apposed cartilage line to a free fragment in the caudal joint compartment [19,28,29]. Intraoperative joint manipulation can alter fragment displacement and the appearance of the cleavage line, underscoring the need for dynamic assessment during the procedure [28,29].

##### 4) Incomplete ossification of the humeral condyle

The intercondylar fracture line can be directly visualized, allowing assessment and irrigation of the fracture plane [30,31]. Associated cartilage changes are seen as linear lesions within the intra-condylar sulcus of the humeral trochlea, ranging from irregular raised ridges, depressed clefts, to bands of chondromalacia [30].

#### Surgical arthroscopy of the elbow joint

##### 1) MCPD

Arthroscopic procedures range from fragment removal to subtotal coronoidectomy with the extent of intervention determined by fragment mobility, lesion location, and cartilage integrity [19,32,33,34,35,36]. ***Free or fixed fragments*** are extracted with grasping forceps or arthroscopic rongeurs, the latter preferred for controlled piecemeal removal without fragment shattering (Fig. 2) [19,32,33]. Embedded or fixed fragments are elevated with a curette, hook probe, or microfracture chisel and removed with caudal force to prevent cranial compartment migration [19,32]. Residual abnormal bone of fixed fragments in the narrow space between the radial head and stable portion of the medial coronoid process, can be accessed using a curved 70° microfracture chisel [19,33]. ***Partial or subtotal medial coronoidectomy*** using power shavers or hand instruments is indicated in cases involving large fragments, free fragments within the cranial compartment, or Grade V chondromalacia and abnormal bone on the fixed portion of the medial coronoid process [19,32,36]. ***Osteophytes*** on the radial head, medial coronoid process, and anconeal process are excised with hand instruments and power shavers to restore joint motion [19,32]. Excision of radial head osteophytes via the medial portal often necessitate medial coronoidectomy for cranial compartment access [19,32,36]. Osteophytes of the anconeal process are removed through caudal portals medial and lateral to the triceps tendon [19,32]. Synovial proliferation, often common, may necessitate bipolar radiofrequency (VAPR) use for effective visualization [19,32,33].

**Fig. 2:**
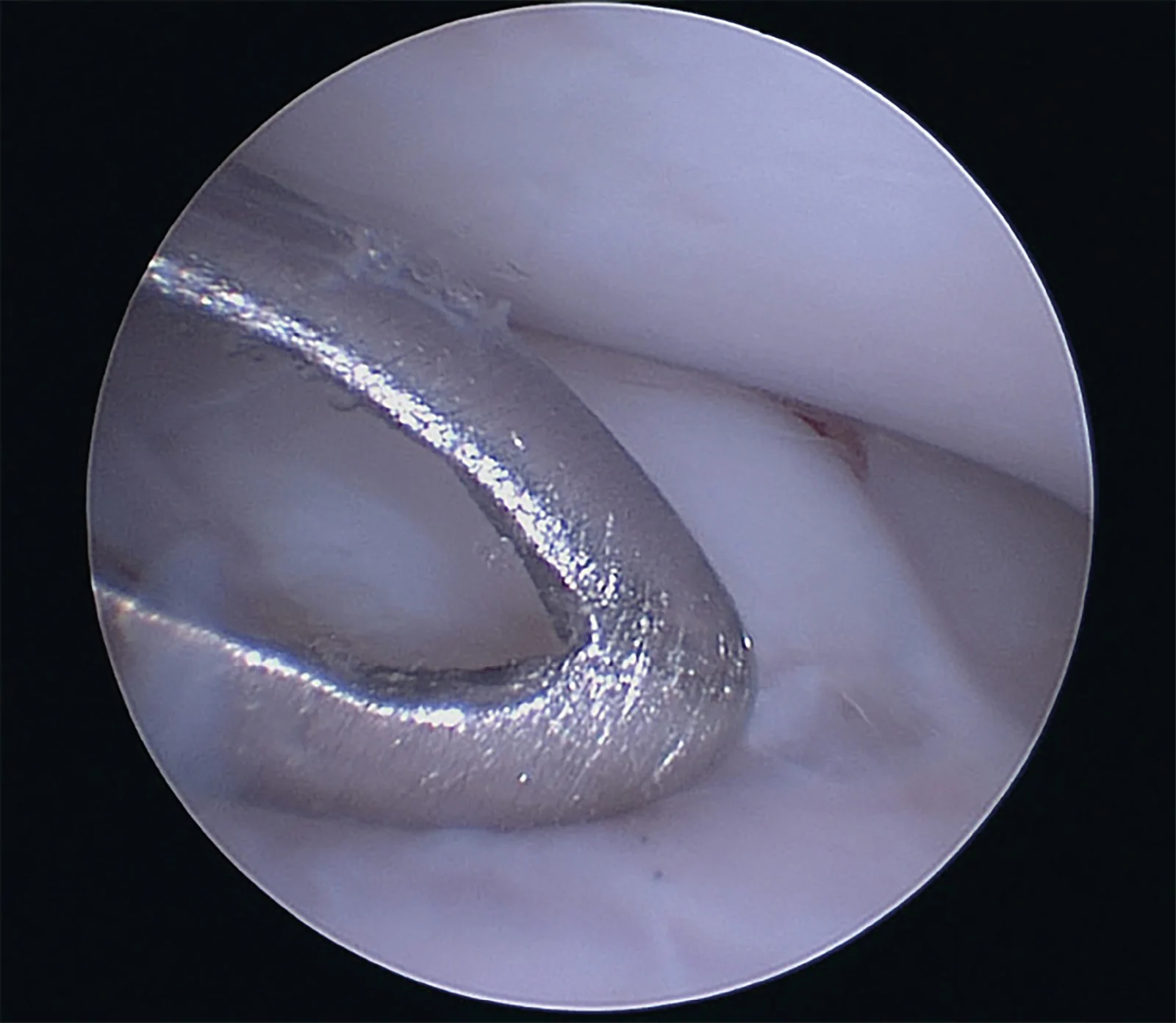
Arthroscopic removal of a free medial coronoid process fragment with arthroscopic rongeurs. To avoid causing iatrogenic damage to the cartilage, rongeur jaws are rotated away from the articular surface before closure around the fragment. In this view, medial is to the left and proximal is up. The arthroscope is introduced through a medial portal and directed craniolaterally, while the rongeurs are inserted through a craniomedial working portal (adapted from McCarthy [19]).

##### 2) Osteochondritis dissecans (OCD)

OCD cartilage flaps are elevated using arthroscopic probes or mosquito hemostats and removed with arthroscopic rongeurs [19,28,37]. Defect margins are curetted to stable cartilage and exposed subchondral bone is evaluated. In cases of avascular subchondral bone, microfractures are created to promote re-vascularization, and in clinical experience, hand instruments are preferred overpower shavers to avoid excessive debridement [27,28,37].

##### 3) UAP

The fragment is approached via a caudomedial tissue tract, elevated from the ulna with a surgical periosteal elevator, and removed with arthroscopic or open surgery rongeurs [38]. Use of a power shaver is generally discouraged due to its inefficiency [19].

##### 4) Intra-articular fractures

Limited open approaches combined with arthroscopic guidance from the medial portal facilitate accurate reduction while minimizing iatrogenic cartilage injury [30,31]. This combined approach improves the precision of screw or pin placement and allows evaluation of associated intra-articular pathology [30,31].

#### Arthroscopy of the shoulder joint

Common indications for arthroscopy for the shoulder joint have been summarized in Table 4 [5].

#### Diagnostic arthroscopy of the shoulder joint

##### 1) Shoulder instability

***Medial glenohumeral ligament lesions*** vary in severity, distribution, and chronicity (acute, chronic, or combined forms) [39,40,41,42]. ***Subscapularis tendon injuries*** present as focal lesions, fiber splitting or complete ruptures [39,40]. Acute lesions show sharply tapered fiber edges, whereas chronic lesions demonstrate vascular proliferation and blunted edges [39,40,41]. ***Supraspinatus tendon*** degeneration, mineralization, and partial ruptures are identified in the craniolateral joint compartment; concurrent capsular rupture permits intra-capsular insertion of a standard 30° arthroscope for lesion visualization [39,40]. ***Lateral labrum avulsion*** presents as fraying, complete detachments, or small osseous avulsion fragments at the labral attachment [39,41]. ***Capsular injuries*** at the medial glenoid margin may mimic medial glenohumeral ligament injuries and often require joint abduction for differentiation [39,41]. Inflammatory changes include hypervascularity, villus or mass-like synovial proliferations and chronic capsular fibrosis [39,40,41,42].

##### 2) Bicipital tendon injuries

Peritendinous synovial proliferation is common, along with partial to complete fiber tears, with rupture distal to the tendon’s origin appearing as longitudinal strands of ruptured or intact fibers [39,43,44]. Avulsion from the supraglenoid tubercle may be present, and avulsion fragments within the tendon can mimic intrinsic tendon lesions [39,43].

##### 3) OCD

OCD lesions of the caudomedial humeral head appear as smooth thin cartilage flaps with indistinct or defined margins [39,45,46]. Detached flaps or fragments may lodge within intra-articular structures or float freely as remodeled arthroliths [45,46]. Adjacent cartilage frequently shows chondromalacia of varying grades depending on chronicity [39,45].

##### 4) Supraglenoid tuberosity avulsion

Cranial glenoid fractures appear as small bone fragments at the biceps brachii tendon origin, along with synovitis and capsular tearing [39,47].

##### 5) Ununited caudal glenoid ossification center (UCGOC)

Lesions present as mobile bone fragments, bony ridges at the caudal glenoid margin, or areas of moth-eaten cartilage [48].

#### Surgical arthroscopy of the shoulder joint

##### 1) Shoulder instability

*Thermal modification* is indicated for medial glenohumeral ligament or subscapularis tendon ruptures only when sufficient residual tissue is present [41,42]. A VAPR device or a standard handpiece with intermittent foot-switch activation to regulate temperature is often utilized [39,41]. Thermally treated tissue requires prolonged recovery; postoperative immobilization with a padded spica bandage for 2–4 weeks is recommended [39,49]. *Arthroscopically guided artificial ligamentoplasty* using threaded sutures, suture anchors, and titanium buttons has shown promising results in cadaveric models [50].

##### 2) Bicipital tendon injury

*Arthroscopic tenotomy* is indicated for isolated partial bicipital tendon ruptures [51,52]. Transection can be performed with an 18-gauge needle, a No.11 scalpel blade, or a VAPR device, the latter preferred for superior hemostatic control [39,52]. Complete transection of all tendon attachments to the bone including the mesotendon, a band of residual synovium, is crucial to prevent post-operative pain from residual tissue stretching and tearing [39,51,52].

##### 3) OCD

Lesion removal is performed through a tissue tract without a cannula [45,46]. A curved hemostat is used to grasp, elevate, rotate, and retract the OCD flap, allowing en-bloc extraction [39,45]. Defect margins are probed, and loose cartilage is trimmed with curettes or shavers [39,45,46]. Exposed avascular/necrotic bone is debrided with hand instruments, and microfractures are created to promote bleeding [45,46].

##### 4) Supraglenoid tuberosity avulsion

Arthroscopic visualization of the fracture plane enables precise implant placements and fragment alignment [31,39,47]. Small intra-articular fragments are removed with rongeurs or hemostats, while irrigation removes blood clots, fibrin, and debris [39,47].

##### 5) UCGOC

Loose fragments are removed with hand instruments or shavers equipped with burrs or aggressive cutting blades [48,49]. The resection site is subsequently probed to confirm complete removal [48,49].

#### Arthroscopy of the radiocarpal joint

Common indications for radiocarpal joint arthroscopy have been summarized in Table 4 [5].

#### Diagnostic arthroscopy the radiocarpal joint

##### 1) Fractures

Arthroscopic visualization of radiocarpal fractures allows assessment of cartilage integrity and intra-articular pathology, thereby facilitating treatment planning and prognostic evaluation [53,54,55,56].

##### 2) Soft tissue injuries

Assessment of the soft tissue injury severity is critical, as severe ligament injuries require open reconstruction or carpal arthrodesis rather than surgical arthroscopy [54,55,56].

#### Surgical arthroscopy of the radiocarpal joint

##### 1) Fractures

Non-structural dorsal slab fractures of the radial carpal bone and dorsal chip fragments of the distal radius are removed with rongeurs [53,54]. Minor dorsal capsular injuries are managed with thermal modification and external splinting [54,57].

### Section 4. Diagnostic and surgical arthroscopy of canine hindlimb

The most common diagnostic and surgical indications for arthroscopy in major canine joints of the hindlimb are summarized in Table 5 [6].

**Table 5:** Major indications and potential complications of arthroscopy in canine hindlimb joints^a^

Joints	Major indications	Potential complications
Hip	Hip dysplasia	Instrument or telescope damage
	Osteoarthritis	Articular cartilage injury
	Hip dislocation	Peri-articular soft tissue injury
	Diagnostic examination	Bone fragment migration
	Septic arthritis	Intra articular bleeding or periarticular fluid accumulation
		Infection
		Nerve injury
Stifle	OCD lesions of the femoral condyle	Instrument or telescope damage
	Injuries of the cranial cruciate ligament	Articular cartilage injury
	Injury of the caudal cruciate ligament	Peri-articular soft tissue injury
	Meniscal examination and treatment	Bone fragment migration
	Osteoarthritis	Intra articular bleeding or periarticular fluid accumulation
	Diagnostic examination	Infection
	Septic arthritis	Nerve injury
Tarsus	OCD	Instrument or telescope damage
	Malleolar and intraarticular fractures	Articular cartilage injury
	Osteoarthritis	Peri-articular soft tissue injury
	Joint instability	Bone fragment migration
	Diagnostic examination	Intra articular bleeding or periarticular fluid accumulation
	Septic arthritis	Infection
		Nerve injury

OCD, osteochondiritis dissecans.

^a^From reference [6].

### Arthroscopy of the stifle joint

#### Diagnostic arthroscopy of the stifle joint

##### 1) Cranial cruciate ligament injuries

***Pronounced villous synovial proliferation*** within the suprapatellar pouch and joint compartments is a characteristic arthroscopic finding in cranial cruciate ligament (CCL) disease [58,59,60]. Synovial reactions may also be present with variable degrees of villous proliferation, while strands of chronic fibrous villous “ghosts” often persist after regression of acute inflammation [58,59,60]. **S*ynovial membrane petechia*** are commonly observed within the suprapatellar area of the proximal trochlear groove, distal patella, and intercondylar fossa [58,59]. ***Vascular pannus*** formation, reflecting abnormal load bearing, is evident on articular cartilage of the trochlear groove, femoral condyles, patella, intercondylar fossa, menisci, and cruciate ligaments, although increased intra-articular pressure may obscure lesion margins [58,59,60]. ***Osteophytes*** appear as flat or irregular bony proliferations or characteristic cobblestone appearance on the medial and lateral trochlear surfaces, distal femoral condyles, and patella [58,61,62]. ***Variable grades of chondromalacia*** may occur throughout the articular cartilage [58,59,60]. ***Acute ligament ruptures*** are characterized by hematoma, sharply tapered ligament ends with intra-fiber striations, and occasional avulsion at ligament origins or insertions (Fig. 3) [58,59]. Partial tears commonly involve ruptured fibers from the medial and lateral aspects of the caudal portion of the distal CCL [58,60]. Minimal injuries manifest as cross striations in intact fibers indicating loss of tension [58,59]. ***Chronic lesions*** exhibit smooth, blunted fiber ends consistent with remodeling, and mixed acute and chronic changes are commonly observed [58,62].

**Fig. 3:**
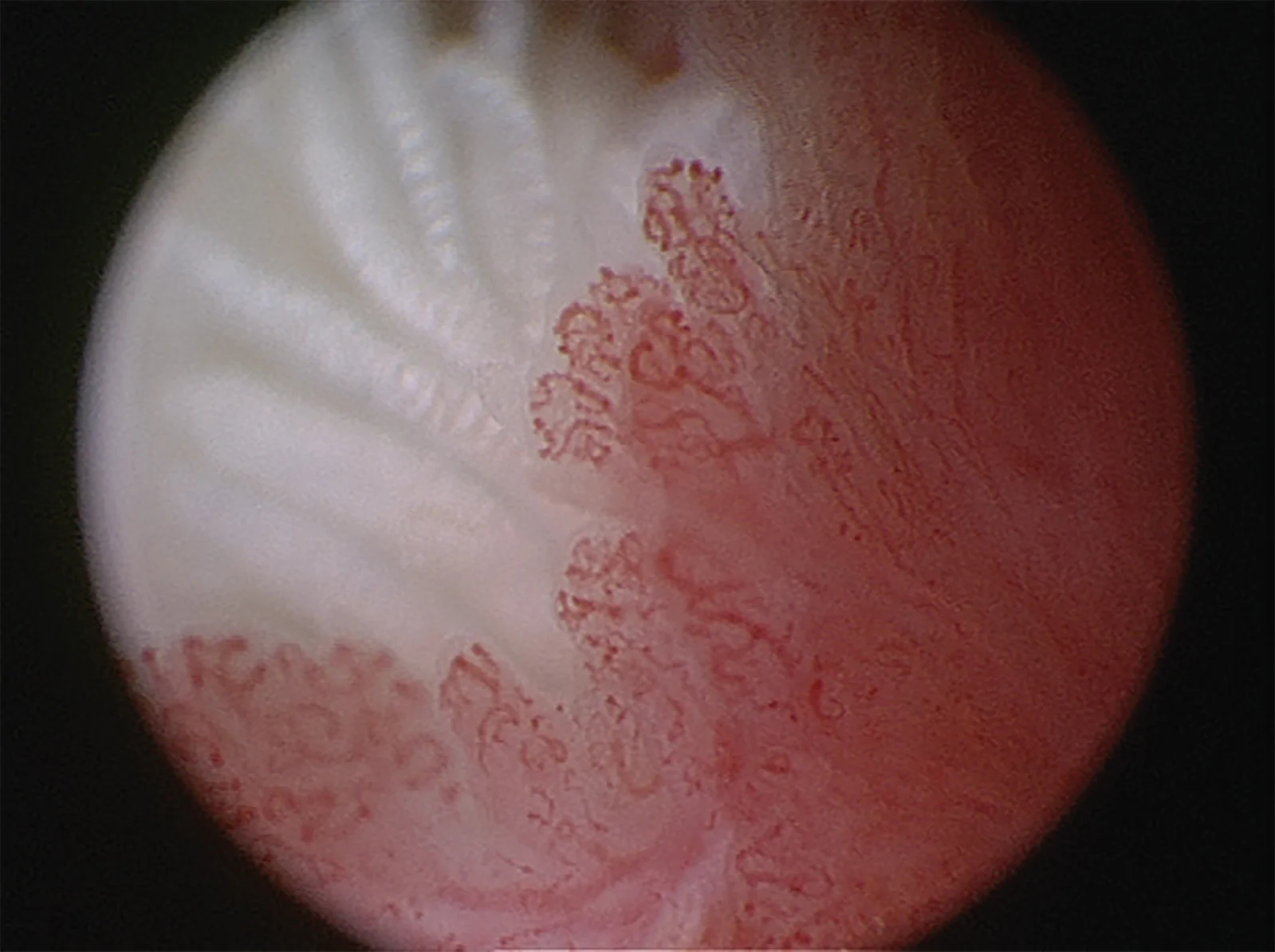
Arthroscopic appearance of an acute cranial cruciate ligament rupture. The lesion is characterized by extensive villus synovial reaction, ruptured ligament remnants with rough margins, and visible cross striations within ligament fibres indicating loss of normal tension. In this orientation, proximal is up and the arthroscope is introduced through a craniomedial portal and directed caudally (adapted from Little et al. [59])].

##### 2) Meniscal injuries

***Medial meniscal injuries*** commonly present as bucket handle or parrot beak tears [63,64,65,66]. Bucket handle tears may be subtle and require displacement into the cranial joint compartment for diagnosis [65,66]. Displacement of the caudal meniscal pole may result in entrapment cranial to the femoral condyles or, in advanced cases, crushing and maceration of the displaced segment [58,63,64]. ***Lateral meniscal lesions*** typically manifest as fraying of the axial margin, with bucket-handle tears occurring less frequently [64]. Recognition of these patterns is clinically relevant, as lesion morphology influences surgical management.

##### 3) Osteochondritis dissecans (OCD)

OCD lesions most commonly affect the medial aspect of the lateral femoral condyle [67,68]. Lesions range from well-defined cartilage flaps, mobile cartilage with irregular margins, to frayed defects or detached fragments within the suprapatellar pouch [58,67]. Cartilage defects range from focal full-thickness losses to extensive erosions [58]. In contrast to CCL-associated pathology, synovial proliferation is generally mild and localized [58].

#### Surgical arthroscopy of the stifle joint

##### 1) Cranial cruciate ligament injuries

Arthroscopic management of CCL injuries includes debridement of partial tears, excision of remnants in complete ruptures, and arthroscopically guided extracapsular stabilization [58,69,70,71]. Partial excision of the cranial fat pad, limited synovectomy, and removal of vascular tissue are performed using radiofrequency devices and shavers to optimize visualization [58,69]. In complete ruptures, frayed ligament ends are excised with a shaver, and residual tissue contoured using radiofrequency ablation to minimize recurrent inflammation [58,69,70]. Stabilization is achieved using arthroscopically guided extracapsular prosthetic ligaments anchored to the caudolateral margin of the lateral femoral condyle [70,71]. Arthroscopically guided intra-articular graft (allografts, autografts, or synthetic grafts) placement, including ultra-high molecular polyethylene implant through the femoral and tibial tunnels have been described; however, the long-term clinical relevance of these techniques remains incompletely defined [72,73,74].

##### 2) Meniscal injuries

Arthroscopic meniscectomy is performed with hand instruments, power shavers, and radiofrequency devices [58,75]. Mobile fragments extending beyond the meniscal rim are excised while preserving intact tissue to maintain hoop stress function [75,76]. ***Bucket-handle lesions*** are resected with duckbill meniscus cutters with margins smoothed using shavers or radiofrequency probes [58,76]. Removal of parrot beak tears and additional meniscal injuries are similarly addressed [58,75]. ***Medial meniscal release*** may be performed with tibial plateau leveling osteotomy (TPLO) to reduce the risk of additional tearing, although its efficacy remains debated [77,78]. Reported techniques include transection of the caudal meniscotibial ligament with a bent VAPR probe or a transverse incision caudal to the ligament attachment under arthroscopic guidance using a scalpel blade, arthroscopic hook knife, or a hypodermic needle [58,77].

##### 3) Osteochondritis dissecans (OCD)

Partially attached cartilage flaps are elevated with a probe and removed using grasping forceps or rongeurs [58,68]. The subchondral bed is debrided with hand instruments, and microfractures are created to stimulate fibrocartilaginous repair [68].

#### Arthroscopy of the hip joint

Common indications for hip joint arthroscopy are summarized in Table 5 [6].

#### Diagnostic arthroscopy of the hip joint

##### 1) Hip dysplasia

Diagnostic arthroscopy for hip dysplasia is primarily used for preoperative evaluation before triple or double pelvic osteotomy [79,80,81]. Assessment focuses on the dorsal femoral head and neck and the central acetabular articular surface, the principal weight-bearing regions after repositioning [79,81]. ***Cartilage wear*** on the medial femoral head dorsal to the fovea capitis - especially medial, dorsal, or dorsomedial lesions – is considered a key negative prognostic indicator [79,81,82]. ***Dorsal femoral and acetabular osteophytes***, appearing as flat or raised bony proliferations along the ventral acetabular rim, can restrict joint abduction post-repositioning [79,80]. ***Soft tissue abnormalities*** include dorsal acetabular labral fraying or avulsion, hemorrhage, partial or complete ligamentum teres rupture or retraction, synovial proliferation around the ligamentum teres and the dorsal capsule, and vascular pannus formation on the articular cartilage [79,80,81,82].

##### 2) Traumatic hip dislocation

Acute traumatic hip dislocation commonly results in tearing of the dorsal joint capsule along with partial or complete ruptures of the ligamentum teres [79,81]. The presence of granulation tissue is indicative of chronicity and may influence treatment planning [79,80].

#### Surgical arthroscopy of the hip joint

##### 1) Hip dysplasia

Although not routine, arthroscopic techniques for managing hip dysplasia have been described [83,84]. Arthroscopically assisted juvenile pubic symphysiodesis involves the application of electrocautery to the pubic symphyseal growth plate to induce localized thermal necrosis of germinal chondrocytes, resulting in premature closure, bony fusion, and concurrent ventrolateral rotation of the acetabula over the femoral heads [83,84]. Despite initial promise, the long-term efficacy and prognostic relevance of this approach require further investigation and careful case selection [83].

##### 2) Traumatic hip dislocation

Arthroscopic management utilizes controlled thermal contraction of the stretched joint capsule and ligamentum teres via a radiofrequency probe [80]. However, long-term outcomes appear to be inconsistent, with frequent post-operative ruptures being reported [80].

#### Arthroscopy of the tibiotarsal joint

Common indications for arthroscopy for the tibiotarsal joint have been summarized in Table 5 [6].

#### Diagnostic arthroscopy of the tibiotarsal joint

##### 1) Osteochondritis dissecans (OCD)

Lesions typically occur on the plantar aspect of the talar ridges and less commonly on the dorsal surface [85,86]. Arthroscopically, lesions appear as cartilage flaps with irregular margins or detached osteochondral fragments [85,86,87]. Pannus formation is common, and cartilage pathology ranges from superficial fraying to full-thickness defects [85,87].

##### 2) Intra-articular fracture

Fracture lines involving the talus or distal tibia are frequently accompanied by articular cartilage damage and free intra-articular fragments [31,85]. The medial and lateral collateral ligaments are evaluated during extension and partial flexion to identify avulsion fragments and assess tissue viability [85].

##### 3) Osteoarthritis

Arthroscopic findings in osteoarthritis resemble advanced OCD, characterized by erosive cartilage wear and symmetrical grooves on opposing articular surfaces, indicative of its chronic state [85,86]. Arthroliths are often associated with cartilage wear and focal subchondral bone exposure [85].

#### Surgical arthroscopy of the tibiotarsal joint

##### 1) Osteochondritis dissecans (OCD)

Arthroscopic treatment aims to reduce pain, control synovial inflammation, and delay osteoarthritic progression [85,87]. Loose fragments are removed with rongeurs or grasping forceps [85,86,87]. Defect margins are debrided with a curette to establish a rim of viable cartilage [87]. Exposed subchondral bone is gently abraded with hand instruments until punctate bleeding is observed [87]. Because plantaromedial lesions are relatively large, aggressive debridement using curettage or a power shaver is avoided to minimize subchondral trauma [85,87].

### Section 5. Potential for application of arthroscopy in wildlife species

Across commonly reported arthropathies of small domestic animals, such as MCPD, CCL disease, and OCD, arthroscopy has demonstrated clinical advantages over arthrotomies, including improved intra-articular visualization and reduced soft-tissue disruption with more precise lesion removal. In this context, minimally invasive surgical (MIS) techniques, including arthroscopy, reduce tissue trauma, promote faster recovery, and minimize disruption of normal anatomy and behavior [88,89]. These benefits are particularly relevant for procedures of wild animals, where prolonged hospitalization is often impractical and may adversely affect clinical outcomes [90].

Despite isolated reports of MIS in both captive and free-ranging mega vertebrates, broader application of MIS, particularly arthroscopy, across wildlife species remains limited [88,89,91]. Arthroscopic procedures have been previously reported in several wildlife species [92,93,94]. Most however, represent single case reports, with only a few studies including cohorts exceeding ten animals, underscoring technical challenges and limited scope of systematic investigation in this field.

Arthropathies have been described in numerous wild species, including the roan antelope (*Hippotragus equinus*), snow leopard (*Panthera uncia*), and wild boar (*Sus scrofa ferus*) [7,89,95,96]. Additional reports describe osteochondrosis-associated arthropathies in free-ranging, juvenile gray wolves (*Canis lupus lupus*) and arthropathies of various causes, in 16 captive raccoon dogs (*Nyctereutes procyonoides*) [7,95,96]. Mycoplasma-associated polyarthritis has also been documented in raccoons (*Procyon lotor*) from the southeastern United States [97]. However, most diagnosis were made post mortem, limiting clinical insight into disease progression, pathophysiology, and therapeutic potential [96,97].

Already well-developed arthroscopic procedures in small domestic animals as previously summarized, have potential for direct adaptation to a range of wild species belonging to closely related taxa, such as raccoon dogs (*N. procyonoides*), wolves, foxes, and other wild canidae and felidae carnivores. Furthermore, micro-diameter arthroscopes, including needle arthroscopes (1–2 mm) described in Section 1, may be suitable for smaller wildlife taxa - particularly small mammals and animals belonging to avian, reptilian, and amphibian taxa - provided that species-specific procedural modifications and standardized protocols are further developed.

In this context, expanded application of minimally invasive arthroscopy offers a valuable opportunity for both surgical management and *in-vivo* investigation of joint disease mechanisms and treatment strategies in wildlife species.

## DISCUSSION

To the authors’ knowledge, this is the first narrative review synthesizing veterinary arthroscopy literature with emphasis on potential wildlife applications. It reviews instrumentation, indications, and techniques for arthroscopy in domestic small animals, primarily dogs, contrasts these with the limited wildlife literature, and integrates dispersed clinical and experimental reports in wildlife species into a framework highlighting translational relevance between domestic and wildlife species.

Arthroscopic application in wildlife species remains limited to isolated case reports, and although arthropathies in wildlife species are increasingly recognized, the predominance of post-mortem necropsy-based diagnoses continues to restrict critical clinical insight into joint diseases pathophysiology, progression, and therapeutic outcomes in wildlife species. In this context, arthroscopy represents not only a MIS approach but also a potential diagnostic modality capable of enabling *in-vivo* evaluation of arthropathies in wild animals.

Key advantages of arthroscopy - minimal invasiveness, shorter procedural and anesthetic duration, faster recovery, and reduced follow up periods - closely align with previously described welfare and management priorities unique to wildlife medicine. These characteristics suggest arthroscopy may offer distinct clinical and welfare benefits in wildlife cases, provided that appropriate translational technical adaptations are implemented.

However, current literature addressing arthroscopic applications in wildlife species remains constrained by inconsistent clinical application, limited case numbers, scarcity of long-term follow-up data, and a predominance of anecdotal evidence. These limitations preclude definitive conclusions regarding clinical efficacy and underscore the need for further structured clinical investigations and the development of species-specific arthroscopic protocols that account for anatomical and behavioral differences.

In the authors’ clinical experience, arthropathies of diverse etiologies—including traumatic, infectious, degenerative, and potentially congenital conditions—are encountered in wildlife species with a frequency comparable to that observed in domestic small animals. Wild avian species frequently present to wildlife centers with subluxations of major wing and leg joints following collisions with man-made structures, while mammals such as raccoon dogs *(Nyctereutes procyonoides)* exhibit a broad spectrum of arthropathies ranging from traumatic, infectious, to degenerative. However, the absence of a systematic and well-established application of arthroscopy in exotic and wildlife species has historically limited their clinical application, often necessitating more conventional approaches involving open arthrotomies along with associated increases in surgical invasiveness, functional impairment, and postoperative morbidity.

Although translational knowledge from small animal arthroscopy may be applicable in select wildlife mammalian species of appropriate size, such as raccoon dogs (*N. procyonoides*), broader clinical implementation remains limited by anatomical diversity among wildlife taxa. These limitations are particularly evident in taxonomically distant groups such as birds and reptiles, where body size, unique anatomy, and corresponding instrumentation constraints restrict routine arthroscopic application. Consequently, conventional open surgical approaches remain the primary diagnostic and therapeutic modalities in wildlife practice despite inherent disadvantages. In avian wildlife species, flight-related anatomical characteristics—including superficially positioned and tightly grouped muscles, tendons, and ligaments around the elbow and carpal joints and the presence of the wing patagium—render open arthrotomies particularly invasive and frequently associated with prolonged rehabilitation, extended hospitalization, and permanent loss of flight capability [98]. Furthermore, prolonged surgical procedures and anesthetic duration related with arthrotomies may increase perioperative risk, as pre-anesthetic assessment is often limited by incomplete clinical history and species-specific physiologic vulnerabilities, including capture myopathy [98,99,100]. Recently developed small-bore needle arthroscopes (1–2 mm diameter) may enable minimally invasive procedures while preserving critical anatomical structures and shortening operative and anesthetic time, thereby improving prognosis in wildlife patients.

In conclusion, arthroscopy is a well-established and highly effective diagnostic and surgical modality in small animal orthopedics, while remaining underutilized yet promising in wildlife medicine. Bridging this gap will require both technical refinement and a conceptual shift toward recognizing arthroscopy as a means of advancing clinical knowledge and improving animal welfare in wildlife species, supported by systematic data collection and outcome-based evaluation.
